# Supplementary material for: Exploration of Li-Organic Batteries Using Hexaphyrin as an Active Cathode Material
Source: Molecules. 2019 Jul 2;24(13):2433. doi: 10.3390/molecules24132433 (PMC6651293; doi:10.3390/molecules24132433)
Supplement: Supplementary file 1 [file molecules-24-02433-s001.pdf]

Exploration of Li-Organic Batteries Using Hexaphyrin as an Active Cathode Material

Ji-Young Shin [1,*], Zhongyue Zhang [2], Kunio Awaga [2] and Hiroshi Shinokubo [1]

[1] Department of Molecular and Macromolecular Chemistry, Graduate School of Engineering, Nagoya University, Furo-cho, Chikusa-ku, Nagoya 464-8603, Japan; jyshinl@chembio.nagoya-u.ac.jp

[2] Department of Chemistry, Graduate School of Science, Nagoya University

* Correspondence: jyshin@chembio.nagoya-u.ac.jp; Tel.: +81-52-747-6771

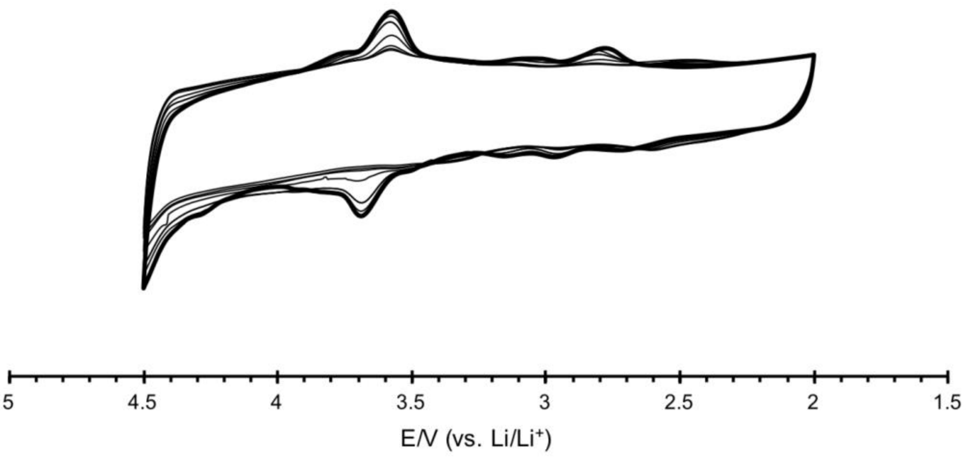

Figure S1. Cyclic voltammogram of Li-[28]hex batteries in 2~4.5 V range set.

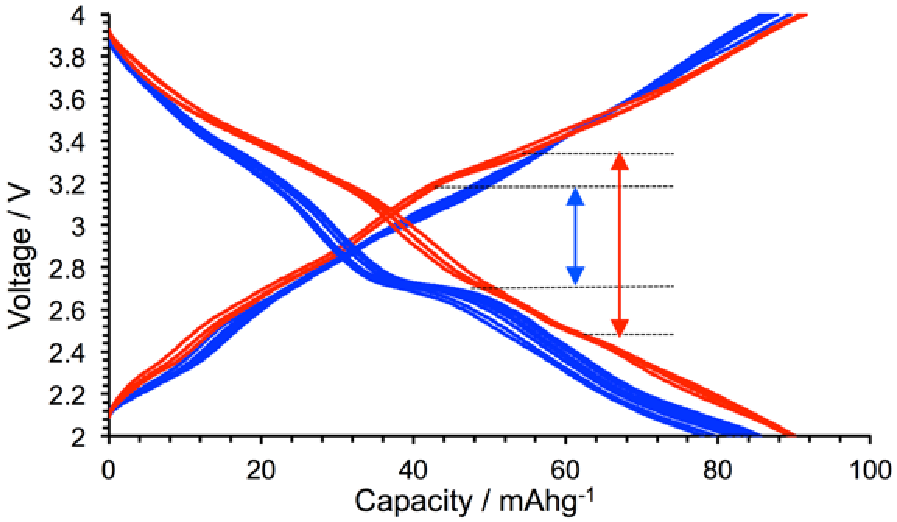

Figure S2. Charge/discharge performances of Li-[28]hex battery: earlier (red) and later (blue) cycles: range = 2 ~ 4 V, number of cycles = 20, and operation current = 0.2 mA.

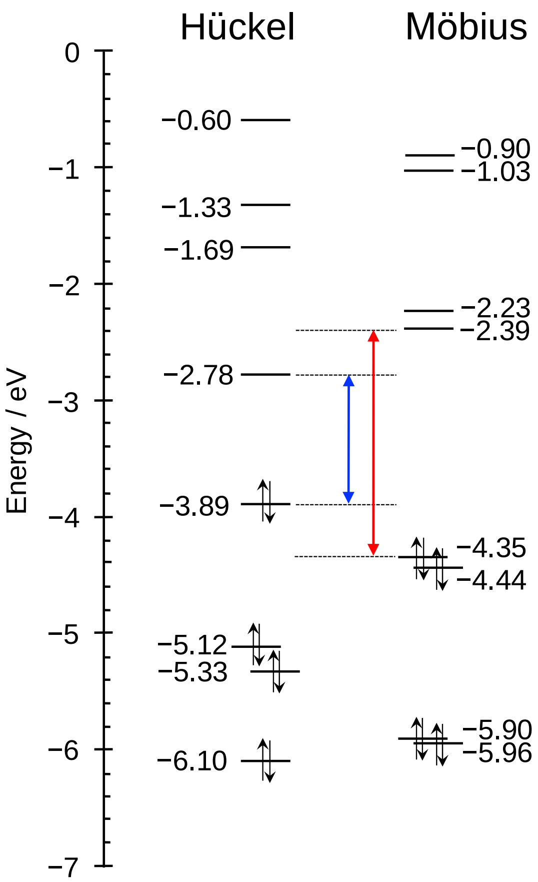

Figure S3. Potential energy diagram for the frontier molecular orbitals of Hückel antiaromatic and Möbius aromatic [28]hexaphyrins.

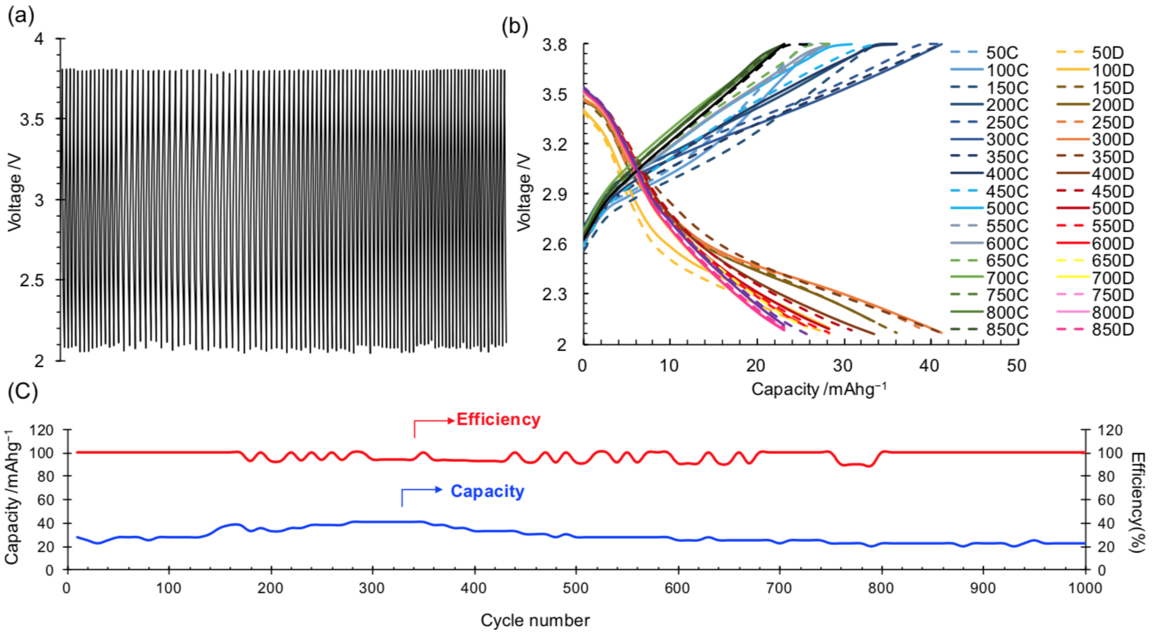

Figure S4. Battery performance Li-[28]hex battery over 1000 cycle: (a) charge/discharge performances (each 10th cycle was projected.), (b) selected charge/discharge graphs (C and D represent charge and discharge curves for the corresponding cycle, respectively.), and (c) capacity and efficiency plots for the 1000 cycle measurements: window's width = 2 ~ 3.8 V, operation current = 3 mA.

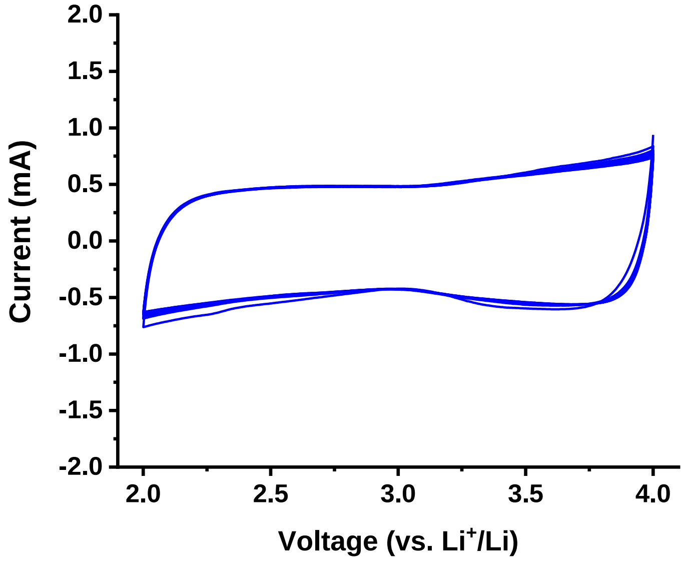

Figure S5. Background CV of pure carbon black for the Figure 3 within the voltage window of 2.0 ~ 4.0 V and a scan rate of 2.0 mV/s.

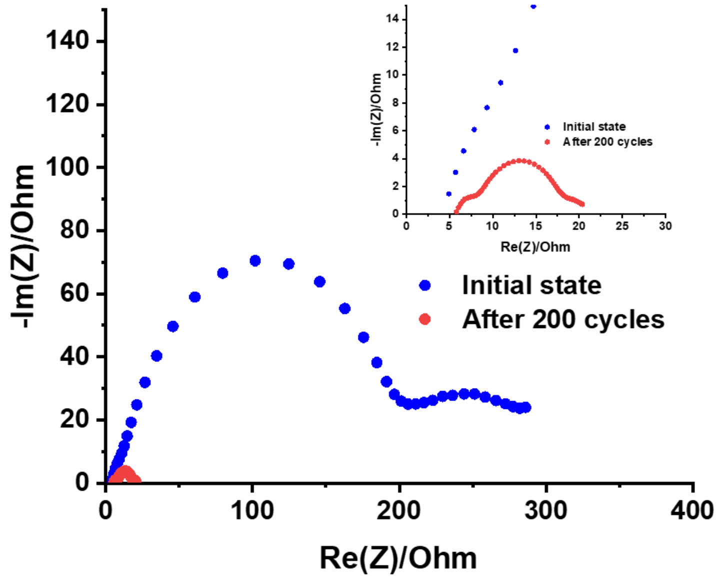

Figure S6. EIS plots before and after 200 cycles of charge/discharge performances. The significantly increased impedance suggests the formation of solid-state electrolyte interface (SEI) layers.
